# The High Prevalence of Oncogenic HPV Genotypes Targeted by the Nonavalent HPV Vaccine in HIV-Infected Women Urgently Reinforces the Need for Prophylactic Vaccination in Key Female Populations Living in Gabon

**DOI:** 10.3390/diseases13080260

**Published:** 2025-08-14

**Authors:** Marcelle Mboumba-Mboumba, Augustin Mouinga-Ondeme, Pamela Moussavou-Boundzanga, Jeordy Dimitri Engone-Ondo, Roseanne Mounanga Mourimarodi, Abdoulaye Diane, Christ Ognari Ayoumi, Laurent Bélec, Ralph-Sydney Mboumba Bouassa, Ivan Mfouo-Tynga

**Affiliations:** 1 Unité des Infections Rétrovirales et Pathologies Associées, Centre Interdisciplinaire de Recherches Médicales de Franceville (CIRMF), Franceville BP 769, Gabon; marden2c@gmail.com (M.M.-M.); ondeme@yahoo.fr (A.M.-O.); engonejordy@gmail.com (J.D.E.-O.); roseannemounanga@gmail.com (R.M.M.); dianeyabdoulaye@gmail.com (A.D.); christayoumi@gmail.com (C.O.A.); tivansdavids2012@gmail.com (I.M.-T.); 2 Ecole Doctorale Régionale (EDR) d’Afrique Centrale en Infectiologie Tropicale, Franceville BP 876, Gabon; 3 Laboratoire de Biologie Moléculaire et Cellulaire (LABMC), Université des Sciences et Techniques de Masuku, Franceville BP 941, Gabon; pamelamoussavoub@gmail.com; 4 Laboratory of Virology, Hôpital Européen Georges Pompidou, Assistance Publique-Hôpitaux de Paris (AP-HP), 75015 Paris, France; laurent.belec@aphp.fr; 5 Faculté de Médecine Paris Descartes, Université Paris Cité, 75006 Paris, France; 6 Institut du Savoir Montfort, Montfort Hospital, Ottawa, ON K1K 0T2, Canada; 7 Department of Family Medicine, Faculty of Medicine, University of Ottawa, Ottawa, ON K1N 6S1, Canada

**Keywords:** high-risk human papillomavirus, cervical cancer assessment, human immunodeficiency virus, cancer antigen 125, Gabonese women living with HIV

## Abstract

**Background/Objectives.** Women living with human immunodeficiency virus (WLWH) have a six-fold higher risk of developing cervical cancer associated with high-risk human Papillomavirus (HR-HPV) than HIV-negative women. We herein assessed HR-HPV genotype distribution and plasma levels of the cancer antigen 125 (CA-125) in WLWH in a rural town in Gabon, in Central Africa. **Methods.** Adult WLWH attending the local HIV outpatient center were prospectively enrolled and underwent cervical visual inspection and cervicovaginal and blood sampling. HIV RNA load and CA-125 levels were measured from plasma using the Cepheid^®^ Xpert^®^ HIV-1 Viral Load kit and BioMérieux VIDAS^®^ CA-125 II assay, respectively. HPV detection and genotyping were performed via a nested polymerase chain reaction (MY09/11 and GP5+/6+), followed by sequencing. **Results.** Fifty-eight WLWH (median age: 52 years) were enrolled. Median CD4 count was 547 cells/µL (IQR: 412.5–737.5) and HIV RNA load 4.88 Log_10_ copies/mL (IQR: 3.79–5.49). HPV prevalence was 68.96%, with HR-HPV detected in 41.37% of women. Among HR-HPV-positive samples, 87.5% (21/24) were genotypes targeted by the Gardasil vaccine, while 12.5% (3/24) were non-vaccine types. Predominant HR-HPV types included HPV-16 (13.8%), HPV-33 (10.34%), HPV-35 (5.17%), HPV-31, and HPV-58 (3.45%). Most participants had normal cervical cytology (62.07%), and a minority (14.29%) had elevated CA-125 levels, with no correlation to cytological abnormalities. **Conclusions.** In the hinterland of Gabon, WLWH are facing an unsuspected yet substantial burden of cervical HR-HPV infection and a neglected risk for cervical cancer. Strengthening cervical cancer prevention through targeted HPV vaccination, sexual education, and accessible screening strategies will help in mitigating associated risk.

## 1. Introduction

Women living with HIV (WLWH) are disproportionately affected by high-risk human Papillomavirus (HR-HPV) infections and cervical cancer, with a risk up to six-fold higher than that of HIV-negative women [1,2]. This disparity is particularly pronounced in sub-Saharan Africa (SSA), where cervical cancer is the second-most common cancer among women and the leading cause of cancer-related death in several countries, especially among WLWH [3,4,5]. According to UNAIDS 2025, SSA was home to 60% of all the 39.4 million adult people living with HIV (PLWH) globally, with women comprising more than half of this population [6]. In 2020, the region accounted for 19.4% of the 604,127 global cervical cancer cases and 22.45% of the 341,831 cervical cancer-related deaths [7]. Moreover, approximately 5% of all cervical cancer cases worldwide are attributable to HIV, with a striking 85.7% of these HIV-associated cases occurring in SSA [2]. The region also includes countries with the highest age-standardized incidence rates of cervical cancer attributable to HIV (exceeding 20 per 100,000 in Eswatini, Lesotho, Malawi, South Africa, Zambia, and Zimbabwe), making African WLWH one of the populations at greatest risk for cervical cancer and related mortality [2,7].

Advances in HIV care across SSA, particularly the widespread roll-out of antiretroviral therapy (ART), have significantly improved life expectancy among PLWH, increasing from 56.5 years in 2010 to 62.3 years in 2024 [6]. However, despite cervical cancer being classified as an AIDS-defining malignancy, routine screening for HR-HPV infection and cervical cancer among WLWH remains largely unavailable in many SSA countries [4,8,9,10]. Screening methods such as HR-HPV DNA testing and Pap smear cytology have proven effective in high-income settings [10]. Nevertheless, their implementation in SSA faces substantial challenges, particularly among WLWH, due to stigma, infrastructural limitations, financial constraints, and a shortage in trained healthcare professionals [10,11]. As a result, the persistently high burden of cervical cancer among WLWH continues to pose a critical public health challenge, threatening to erode the survival gains, achieved through ART and continued care [12,13]. There is an urgent need to develop and scale up more accessible and effective screening and prevention strategies tailored to WLWH in SSA.

Cervical cancer is primarily caused by persistent genital infections with one of the 13 HR-HPV genotypes, notably HPV-16 and HPV-18, which are responsible for over 70% of cases in the general population, and up to 90% among WLWH [14]. The remaining HR-HPV types (HPV-31, HPV-33, HPV-39, HPV-45, HPV-51, HPV-56, HPV-58, HPV-59, and HPV-68) also contribute substantially to the cervical cancer burden in SSA [14]. Despite regional variation in genotype distribution, HPV-16, HPV-35, HPV-52, and HPV-58 consistently rank among the most prevalent in Central Africa, in both the general population and WLWH [15,16,17,18,19,20,21,22,23,24]. Several of these genotypes are targeted by current multivalent HPV vaccines such as Gardasil^®^ (quadrivalent) which covers HPV-16, HPV-18, and low-risk (LR)-HPV types HPV-6 and HPV-11, while the nonavalent Gardasil-9^®^ provides a broader spectrum protection and covers five additional oncogenic types (HPV-31, HPV-33, HPV-45, HPV-52, and HPV-58) [14,25]. Many low-income SSA countries, supported by international NGOs such as GAVI, have introduced HPV vaccination programs primarily using the quadrivalent vaccine, with national campaigns focusing mainly on adolescent girls aged 9–14 years according to WHO recommendations, often through school-based delivery [26,27]. However, countries non-eligible for GAVI support such as Gabon has not yet implemented any HPV vaccination programs [28]. Nevertheless, the high prevalence of non-vaccine oncogenic genotypes, particularly HPV-35, especially in WLWH, may compromise the vaccine effectiveness in this high-risk population, as vaccine cross-protection against non-vaccine types is only limited and tends to wane over time [14,21,23,27,29,30,31,32,33]. Improving HPV vaccination coverage in WLWH in SSA is critical, but must be coupled with innovative monitoring strategies based on novel biomarkers for identification of early events in the pathogenesis of cervical cancer. This would enable better surveillance of WLWH infected with diverse HR-HPV genotypes and at higher risks of disease progression [3,21].

Given the elevated risk of genital cancer among WLWH, especially in underserved rural settings, there is a critical need for accessible and cost-effective biomarkers to aid in early detection. The cancer antigen 125 (CA-125) is a high-molecular-weight glycoprotein of the mucin family that has been extensively studied as a tumor marker in other non-HPV-associated gynecological cancers such as ovarian and endometrial cancers with demonstrated utility in monitoring disease progression, treatment response, and differentiating malignant from benign lesions [34,35,36,37,38,39,40]. Otherwise, published data on CA-125 levels in women from Central African countries, including Gabon, are currently lacking. Thus, investigating CA-125 levels in this context may help to assess its possible value as a complementary, less-invasive screening tool for non-HPV-associated gynecological cancers, especially in remote areas where access to standard sanitary facilities for HPV-based screening technologies is limited.

Located on the west coast of central Africa, Gabonese people have recently faced a generalized HIV epidemic, with adult prevalence consistently ranging from 3 to 4% over the recent decades. A recent national survey reported higher HIV prevalence in women, particularly in rural areas (6.0% versus 4.7% in urban settings), and underscoring the regional disparity in the HIV burden [41]. These data also suggest a higher cervical cancer risk for WLWH in rural areas in Gabon. The Gabonese Ministry of Health implemented a program for cervical cancer screening and awareness so-called “*Octobre Rose*” that targets women of childbearing age in the general population [19,42]. However, among WLWH who are at higher risk for cervical cancer, especially those living in hard-to-reach rural areas, no comparable initiative has yet been implemented.

The aim of this pilot exploratory study was to characterize the distribution of HR-HPV genotypes in hard-to-reach WLWH attending the local HIV outpatient treatment center in Koulamoutou, a rural city in Middle Eastern Gabon.

## 2. Materials and Methods

### 2.1. Study Design, Population Enrolment and Specimen Sampling

**Figure 1:**
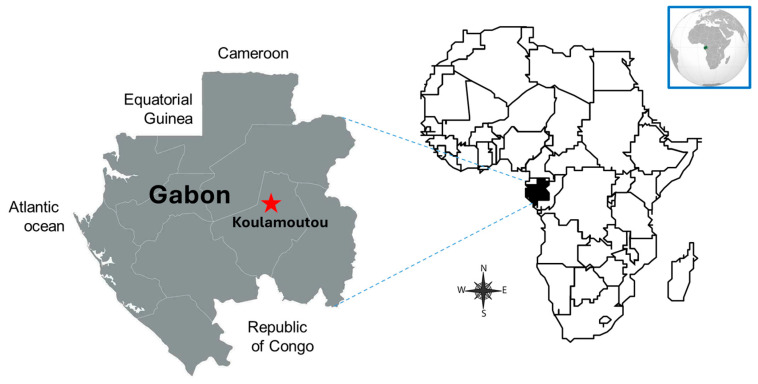
Inclusion site in Koulamoutou, a rural area in Middle Eastern Gabon. The city of Koulamoutou is the capital city of the Ogooue Lolo province in rural Middle Eastern Gabon.

This pilot and cross-sectional study was carried out from 12 to 23 June 2023 among WLWH attending a HIV outpatient treatment center in Koulamoutou, the capital city of the rural Ogooue Lolo province in Middle Eastern Gabon (Figure 1). The designed center is the reference treatment facility for people with HIV living across the towns and villages of the province. The objective and technical procedures of the study were thoroughly explained to adult WLWH, and they voluntarily decided to participate in the study. All participants were sexually active WLWH, aged above 18 years old, and regularly attending the reference HIV outpatient treatment center. Women who had undergone hysterectomy and those who were pregnant, lactating, or menstruating were excluded from the study.

Participations were selected upon receiving both signed informed consent forms and completion of a standardized study questionnaire. We set a convenient sample target size of 60 participants for this study to validate preliminary outcomes before further investigations. Sociodemographic, economic, sexual, and reproductive health data of the participants were collected by a health practitioner at the inclusion using a standardized questionnaire. Following completion, participants underwent a downstaging “naked-eye” visual examination of the tissular aspect of the cervix using a gynecological examination lamp after insertion of a speculum. Any observed abnormalities were documented. Cervical appearances ranged from healthy-looking tissue, classified as normal, to signs of inflammation or ectropion, which were classified as abnormal. Then, the practitioner collected a cervicovaginal sample for HPV DNA detection and genotyping in liquid medium (ThinPrep^®^; Hologic Bedford, MA, USA) using a cervical brush (Cervex-Brush, Hologic, Bedford, MA, USA). Thereafter, a 10 mL venipuncture blood sample was collected in an Ethylenediaminetetraacetic acid (EDTA)-containing tube from each woman. The blood tubes were then centrifuged at 3000 rpm for 10 min before plasma and the white blood cells layer were separately collected in 2 mL conical tubes and stored at a −80 °C facility at the HIV outpatient center. All frozen plasma, white blood cells and cervicovaginal samples were then placed in icepacks and transferred to the laboratory facility located at the Retroviral Infections and Associated Pathologies Unit (UIRPA) of the *Centre Interdisciplinaire de Recherches Médicales de Franceville (CIRMF)* and stored at −80 °C before further technical processing.

### 2.2. Blood Plasma HIV-1 RNA Load Quantification

The HIV-1 RNA load was quantified in 1 mL of plasma using the Xpert^®^ HIV-1 Viral Load kit from Cepheid^®^ according to the manufacturer’s instructions (Cepheid, Sunnyvale, CA, USA). The Xpert^®^ HIV-1 Viral Load assay, based on GeneXpert^®^ technology, automates the entire testing process, encompassing RNA extraction, purification, reverse transcription, and real-time cDNA quantification, within a single, fully integrated cartridge.

### 2.3. HPV Detection, Sequencing, and Genotyping

Total genomic DNA was extracted from exfoliated cells using the DNeasy Blood and Tissue kit (Qiagen, Valencia, CA, USA) following the manufacturer’s instructions. The detection of HPV DNA was carried out from the extracted genomic DNA using a nested conventional polymerase chain reaction (PCR). The first PCR used the degenerate consensus primers pair MY09/MY11 targeting and amplifying a 450 bp of the HPV L1 gene. The PCR settings were adapted from our previous reports [19,43]. Briefly, a 50 μL reaction mix was constituted with 5 μL of extracted DNA, 1X PCR buffer (Invitrogen, Carlsbad, CA, USA), 2 mM MgCl_2_, 0.2 mM of each deoxynucleotide triphosphate (dNTP), 0.3 μM of each primer MY09/MY11, and 0.02 U of Taq DNA polymerase (Invitrogen, CA, USA). The thermal cycling was as follows: 10 min at 95 °C, 40 cycles of 45 s at 95 °C, 45 s at 55 °C, and 40 s at 72 °C, with a final extension step at 72 °C for 8 min.

The second step of PCR used the GP5+/GP6+ primers that amplify a 150 bp sequence within the 450 bp amplicon previously amplified. Then, 5 μL of the MY09/MY11-based PCR products were added in 45 μL of the nested PCR mix composed of 1× PCR buffer, 2 mM MgCl_2_, 0.2 mM of each dNTP, 1 μM of each primer GP5+/GP6+, and 0.02 U of Taq DNA polymerase. Samples positive for HPV with the conventional nested PCR were sent to the MACROGEN EUROPE BV (Meibergdreef, Amsterdam, Netherlands) for sequencing using the primers pair GP5+/GP6+. Obtained sequences were aligned and analyzed using the MEGA 9.1 software (https://www.megasoftware.net/ (accessed on 13 August 2024)) and mapped against HPV reference sequences using the BLAST 2.16.0 application (http://www.ncbi.nlm.nih.gov/blast/ (accessed on 13 August 2024)) from GenBank (NCBI, National Institute of Health, Bethesda, MD, USA) to identify the corresponding HPV genotypes as described previously [20,44]. It is noted that only the sequences generated from the primer sense (GP5+) were used to determine HPV genotypes. Those generated from the primer antisense (GP6+) were uninterpretable and were excluded from the analysis.

### 2.4. Cancer Antigen (CA) 125 Level Measurements

The CA-125 quantification was carried out in 200 μL of plasma using the VIDAS^®^ CA-125 II assay from BioMérieux (bioMérieux S.A., Marcy l’Étoile, France), according to the manufacturer’s instructions. This is an automated quantitative test performed on VIDAS instruments, which measures the epithelial ovarian tumor-associated reactive antigen (also called CA-125) based on the enzyme linked fluorescent assay principle with the limit detection ranged from 4.00 to 600.00 U/mL. The cut-off value of CA-125 plasma levels ≤ 35 U/mL was considered as the normal upper limit or threshold as conventionally admitted [34,35,36].

### 2.5. Statistical Analysis

Data were organized and entered into an Excel spreadsheet before analysis using the standard statistical software DATAtab version 1 (Graz, Austria; https://datatab.net (accessed on 5 November 2024)). Quantitative variables were summarized using means and standard deviations (SD), and categorical variables as proportions with associated 95% confident interval (CI). Association between categorical variables were tested using Pearson’s χ2 or Fisher’s exact test, while continuous variables were analyzed using the Mann–Whitney U-test or Kruskal–Wallis’s rank sum test. A two-tailed *p*-value < 0.05 was considered statistically significant. Logistic regression using univariate and multivariate analyses were performed to identify the potential determinants associated with cervical cytology and HPV outcomes. The crude odds ratio (cOR) from the univariate and adjusted odds ratio (aOR) from the multivariate analysis were calculated and reported along with their 95% CI. Variables with cOR and aOR strictly greater than “1” with a *p*-value less than 0.05 were considered a significant risk factor.

### 2.6. Ethics Statement

All participants were aged 18 years and older, and signed the informed consent form of the study prior to enrollment. The study was conducted in accordance with the Declaration of Helsinki and approved on 10 March 2013, by the National Ethics committee of Gabon, registered as PROT N°0010/2013/SG/CNE.

## 3. Results

### 3.1. Study Population Characteristics

During the period of the study (12 to 23 June 2023), 58 adult women (median age: 52 years) attending the HIV outpatient treatment center of Koulamoutou in the Middle Eastern Gabon were recruited on a voluntary basis. The detailed sociodemographic and clinical characteristics of these women are listed in Table 1. Most of the women were over the age of 30 years, with no discrepancy among age groups and the densest was that made of quinquagenarians with 36.2%. Half of these women (50.0%) were single, about a third made of women (32.76%) engaged in a couple relationship, and a minority were widowed (15.52%). The majority of women (*n* = 31; 53.44%) did not reach high school and tertiary education, while only one woman (1.72%) had a University degree. A little less than a quarter (24.14%) of these women were employees with a regular monthly salary, one-third (32.76%) practiced subsistence farming, and 39.66% were unemployed. Regular alcohol consumption was frequently reported in that rural population (48.28%), yet only five women (8.62%) were active smokers. The majority of women (*n* = 41; 70.69%) had their first sexual intercourse between 15 and 20 years old, while a minority (*n* = 9; 15.52%) declared the start of sexual activity before 15 years old, and a lesser proportion (*n* = 4; 6.90%) after 20 years old. Moreover, most of these women (*n* = 33; 56.90%) declared having had sex with more than five partners, while only two women (3.45%) reported to have a unique lifelong sex partner. In addition, only few of these women (15.52%) were using medical contraceptives, while almost two-third (60.34%) had not used it. All the participants have been pregnant at least once in their lifetime, with half (51.72%) reporting 5 to 9 pregnancy episodes, 29.31% had 1 to 4 pregnancies, and 17.24% had experienced over 10 pregnancies. Of these women, only one (1.72%) reported to never having given birth, while half (51.72%) reported 1 to 4 episodes of parity, more than one-third (37.93%) reported 5 to 9 episodes, and only 8.62% had experienced over 10 episodes of parity. Most of the women (82.76%) did not experience any miscarriage, while one-fifth (15.51%) failed to give birth at least once, with 13.79% reporting up to four episodes. Voluntary abortion was also regularly reported, with 58.62% reporting up to four abortions, 5.17% over five episodes, while 34.48% declared to never have undergone voluntary abortion.

History of STI other than HPV was also infrequent in the women, as two-thirds (62.07%) of them reported never having been tested positive for any of the screened pathogens, and only a minority (5.17%) declared to having been tested positive for a STI before. On the other hand, more than half (56.90%) of the women reported to already having been screened for HPV at least once, and 43.10% reported to never undergoing this test before (Table 1).

Regarding the cytological aspect of the cervix, most of the participants showed normal cytology (*n* = 36; 62.07%) and only one-third (36.21%) presented cytological abnormalities in their cervix uteri (Table 1).

The HIV parameters of the study population are reported in Table 2. The included women were living with HIV for almost a decade (median time since first HIV diagnosis: 7 years) and they were all receiving an ART regimen with TDF-3TC-DTG (*n* = 57; 98.28%) or TDF-3TC-ATV/r (*n* = 1; 1.72%). Although almost half (44.83%) of these women had a CD4 T cell count above the threshold for immuno-depression (CD4 counts < 500 cells/μL), the remaining ones (34.48%) harbored CD4 T cell counts indicating mild (13.79%), moderate (5.17%), severe (10.34%), and extremely severe (5.17%) immuno-depression. Regarding HIV RNA plasma load, all the participants with available data showed detected HIV RNA load. Of those, only 10.34% had less than 1.7 Log_10_ RNA copies per ml, while most of the others carried low to moderate (17.24%), high (8.62%), very high (15.51%), and extremely high (32.76%) HIV RNA load in their plasma samples. It is noted that for a few participants, we failed to quantify the CD4 T cell count (*n* = 12; 20.69%) and the HIV RNA plasma load (*n* = 9; 15.52%) (Table 2).

### 3.2. Prevalence of HPV Detection and Genotypes Distribution

**Figure 2:**
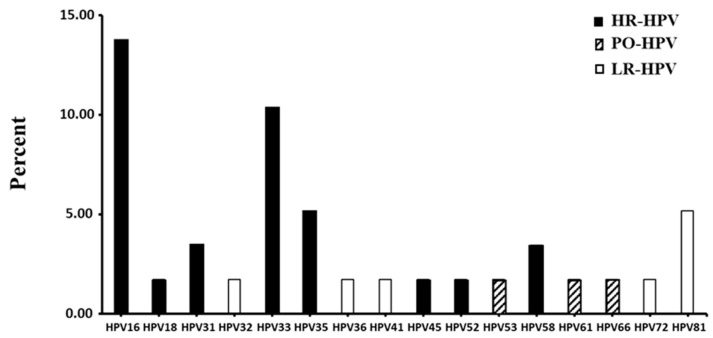
Distribution of HPV genotypes in genital secretions among 58 women living with HIV in Middle Eastern Gabon. Black bars represent HR-HPV types, gray bars represent potentially oncogenic types (PO-HPV), and white bars are for low-risk (LR)-HPV.

The HPV results are presented in Table 3 and Figure 2. Out of the 58 women tested for HPV DNA with the consensus nested PCR method, 40 were positive, corresponding to a total HPV prevalence of 68.96%. Of these 40 women, 29 were successfully sequenced and genotyped, while 11 failed to provide interpretable genomic sequences. The proportions related to HPV characterization profile are provided from the total 58 included women. Accordingly, the total prevalence of HPV infection was 68.96% and HR-HPV prevalence was 41.37%, with 87.5% (21/24) of HR-HPV-positive samples carrying oncogenic genotypes targeted by the Gardasil vaccine, while only 12.5% (3/24) of HR-HPV detected were non-vaccine types. The prevalence of low-risk genotypes as well as genotypes considered as possibly carcinogenic was 13.79% (Table 3). As presented in Figure 2, the vaccine HR-HPV genotypes HPV-16 (13.79%) and HPV-33 (10.34%) were the most detected genotypes, followed by the non-vaccine oncogenic HPV-35 and the low-risk HPV-81 (5.17%), as well as the vaccine oncogenic types HPV-31 and HPV-58 (3.45%). The other identified HPV types were detected only once. Interestingly, apart from HPV-35, no other non-vaccine HR-HPV was detected in this study population. No significant difference in terms of prevalence of HPV infection was observed between the different age groups, though HPV infection was less frequent in women aged 30 to 39 (36.36%), and those aged 40 to 49 were the most frequently positive (46.15%).

### 3.3. CA-125 Plasma Levels and Associated Risk for Detection of Cervical Cytological Abnormalities

A subset of 35 out of 58 included participants (60.34%) underwent CA-125 plasma level quantitation. Thus, all the analyses regarding CA-125 and the risk assessment for cervical cytological abnormalities were carried out only on the tested 35 participants. We found that more than three-quarters of the women (*n* = 30; 85.71%) had plasma levels of CA-125 below the threshold value for normal level of CA-125 in blood (<35 U/mL) and were considered as negative for this marker. In contrast, only five women (14.29%) showed CA-125 levels above the cut-off value and were considered as positive for this marker. No association was observed between the plasma level of CA-125 and cervical cytological abnormalities (*p* = 0.17). No gynecological cancers, such as ovarian and endometrial cancers, could be evidenced in women with elevated blood levels of CA-125.

### 3.4. Potential Risk Factors Associated with HPV Outcomes by Logistic Regression Analyses

To identify factors that determine the genital HPV outcomes in HIV-positive Gabonese women living in Koulamoutou, sociodemographic and clinical characteristics were calculated in univariate and multivariate logistic regression analyzes. Results are presented in Table 4. Overall, no significant risk factor was highlighted by the logistic regression analysis.

## 4. Discussion

We herein report on HR-HPV prevalence and genotype distribution in a cohort of adult WLWH attending a HIV outpatient care unit in a rural area in Middle Eastern Gabon. To our knowledge, this is the first study addressing genital HR-HPV characterization in WLWH in Gabon. In addition, we also explore the relevance of plasma levels of CA-125 as a potential biomarker for non-HPV-associated gynecological cancers in this convenient sample of WLWH. Overall, we recorded a high prevalence of HR-HPV (41.37%), corresponding mainly to genotypes targeted by the Gardasil-9^®^ vaccine (87.5%, 21/24), especially HPV-16 (13.79%) and HPV-33 (10.34%), the first and the second-most detected genotypes, respectively. HPV-35 was the only oncogenic type not covered by HPV vaccines which was detected in these women. On the other hand, most of the women showed normal cervical cytology (62.07%). Furthermore, no correlation was observed between plasma levels of CA-125 and cervical cytological abnormalities, and elevated blood levels of CA-125 was not associated with ovarian or endometrial cancers. Thus, the high prevalence of HPV-16, the most carcinogenic genotype, observed in this population highlights the hidden yet significant risk of cervical cancer faced by WLWH in rural areas of Gabon. Extending the national cervical cancer screening program to include WLWH in remote and/or rural areas of Gabon is essential for improving the early detection and prevention of HPV-16-associated cervical lesions and cancers.

We observed a relatively high prevalence (41.37%) of genital HR-HPV infection among these Gabonese WLWH. This finding aligns with the well-established association between HIV infection and increased susceptibility to HR-HPV. Indeed, a recent systematic review [21] including 17 studies conducted over the last two decades across SSA countries supports our observation, reporting HR-HPV prevalence rates among WLWH ranging from 37.7% [45] to 79.1% [46]. Additionally, a recent study from the neighboring Republic of Congo, which included 18% of HIV-positive participants, reported an even higher genital HR-HPV prevalence of 83% among WLWH [23]. To contextualize our findings within the broader HR-HPV epidemiological context of Gabon, it is important to note that no prior data exist on genital HR-HPV infection among WLWH in the country. The HR-HPV prevalence (41.37%) observed in our study is higher than the 24.8–25.4% reported among HIV-negative Gabonese women with normal cervical cytology [42,43]. Interestingly, this prevalence was similar to that seen among HIV-negative Gabonese women with precancerous cervical lesions (44.6%) [20], and not far below the much higher rates (92.4–94.6%) observed in women with invasive cervical cancer [18,19,22]. Thus, our findings highlight the elevated burden of genital HR-HPV infection among WLWH in rural Gabon and emphasize the urgent need for tailored screening and prevention strategies in this high-risk population.

HPV-16, the genotype responsible for approximately 61.7% of the global burden of invasive cervical cancer [14], was the most frequently detected HR-HPV type in our study population. This finding aligns with HR-HPV genotype distribution patterns commonly reported among WLWH across the SSA region [21], and is consistent with the HPV epidemiological landscape observed in the general population in Gabon, where HPV-16 is frequently the predominant genotype in both women with normal cervical cytology and those with cervical cancer [18,19,20,22,42,43]. Collectively, these findings suggest that HPV-16 is widely circulating in Gabon, not only in the general population but also among WLWH from remote areas, underscoring a neglected yet elevated risk of cervical cancer in this highly vulnerable group. Indeed, cervical cancer is currently the second leading cancer among women in Gabon [47], and WLWH are estimated to have up to a six-fold higher risk of developing cervical cancer compared to HIV-negative women globally [1,2]. Despite this burden, Gabon lacks a national HPV vaccination program [28,47]. Introducing broad-spectrum vaccines such as Gardasil-9^®^ which targets nine HPV genotypes including seven HR-HPV types (HPV-16, HPV-18, HPV-31, HPV-33, HPV-45, HPV-52, and HPV-58) could significantly reduce the circulation of HPV-16 and other oncogenic genotypes prevalent in the region [48]. In the long term, this may contribute to a meaningful reduction in HPV-related cervical cancer incidence [14,48]. The potential benefits of such a broad-spectrum vaccine may a priori be particularly impactful among WLWH, as our study revealed high frequencies of other HR-HPV types such as HPV-33, HPV-31, and HPV-58, which are only included in the nonavalent Gardasil-9^®^ formulation. These findings support the relevance of transitioning to Gardasil-9^®^ in future vaccination strategies in Gabon, especially in high-risk population such as WLWH. However, vaccination alone may not be fully effective in these HIV-positive women unless it is implemented as part of a comprehensive prevention strategy that also includes routine screening with primary HPV DNA testing [49]. This is especially relevant given the high prevalence in our study of HR-HPV types not covered by any of the current vaccines, particularly HPV-35. Although some studies suggest limited cross-protection against HPV-35, mainly with the bivalent vaccine, this protection is inconsistent and likely reduced in immunocompromised individuals such as WLWH [50,51,52,53]. HPV-35 has also been identified at high frequencies among WLWH in several other SSA countries, including South Africa and across the continent. For instance, a multi-country study published in 2016 found that HPV-35 was disproportionately more frequent in WLWH diagnosed with invasive cervical cancer compared to their HIV-negative counterparts [29]. In Eastern and Southern Africa, subsequent studies have also shown that HPV-35 ranks among the most prevalent HR-HPV genotypes in WLWH with cervical intraepithelial lesions, further emphasizing its significance in this population [31,32]. Likewise, data from Nigeria also revealed frequent detection of HPV-35 in HIV-negative women with cervical cancer and among WLWH, highlighting similar concerns in West Africa [54,55,56].

Strengthening cervical cancer screening programs for WLWH through regular primary HPV DNA testing remains essential [49]. However, for WLWH residing in remote, resource-limited rural areas, such as those represented in our study, alternative low-cost but effective approaches like visual inspection with acetic acid (VIA) and/or Lugol’s iodine (VILI) can facilitate the early detection of cervical lesions. These methods have demonstrated strong effectiveness, ease of implementation, and contextual suitability in SSA [57]. Moreover, VIA/VILI have been widely used for nearly a decade during the annual national cervical cancer screening campaign “Octobre Rose” in Libreville, the capital of Gabon [23,42]. However, in the absence of VIA/VILI services in rural central Gabon, we employed a downstaging “naked-eye” visual examination of the cervix to identify cervical abnormalities. Using this low-sensitivity method, we observed a relatively low rate of cytological abnormalities (36.2%) in our study population. These findings are yet consistent with another report from Gabon that used Pap smear cytology and highlighted a 28.7% prevalence of precancerous cervical lesions in a cohort of 115 WLWH in Libreville [58]. Furthermore, systematic reviews and meta-analyses across the SSA region have reported slightly lower prevalence rates among WLWH, ranging from 15.3% to 25.6% [59,60,61]. Although our findings were obtained using a less sensitive screening method, they still point out a substantial burden of cervical abnormalities among WLWH in the rural areas of Gabon, comparable to, or even higher than, rates reported in urban settings. These results underscore the urgent need to expand access to effective cervical cancer screening tools in underserved regions, particularly for high-risk populations such as WLWH.

In this pilot study, most women showed normal circulating levels of CA-125. Elevated levels of CA-125 was not associated neither with cervical dysplasia, nor with non-HPV-related gynecological cancers. These findings should be interpreted with caution given the small sample size and the exploratory nature of our study. Overall, while our data suggest limited value for CA-125 in detecting early gynecological cancers in WLWH, further studies involving larger and more diverse cohorts are needed to fully evaluate its potential role, either alone or in combination with other biomarkers, in this high-risk population.

Our study has several limitations that need to be considered, notably the small sample size that might have reduced the possibility to pinpoint a potential benefit of elevated CA-125 plasma levels for detecting early genital abnormalities in WLWH. Moreover, this may also have underestimated the impact of the potential predictors of HR-HPV infection in that at risk subpopulation. Due to the limited resources in the rural HIV care unit of Koulamoutou in the center of Gabon, the very low-sensitive and specific method downstaging “naked-eye” examination was used for detecting cervical abnormalities in our study. This could also have led to an overestimation of cervical cytological abnormalities with no real clinical implication in the context of cervical cancer. Finally, for a little more than a quarter (27.5%; 11/40) of women positive for HPV DNA with the MY09/11- and GP5+/6+-based nested PCR assay, we failed to determine the corresponding genotype with the sequencing method. In the context of remote and restrained resources setting, commercially available multiplex HPV testing kits would be a better option.

## 5. Conclusions

Our findings reveal a substantial burden of HR-HPV infection, particularly high-risk genotype HPV-16 among WLWH in rural Gabon, underscoring a neglected risk for cervical cancer in this underserved population. The absence of correlation between CA-125 plasma levels and cervical abnormalities suggests limited utility for this biomarker in early screening for gynecological cancers in that population. Strengthening cervical cancer prevention through targeted HPV vaccination and accessible screening strategies such as commercially available multiplex HPV testing and VIA/VILI remains urgently needed.

## Figures and Tables

**Table 1:** Characteristics of the women living with HIV included in the study.

	Study Participants (*n* = 58)
**Characteristics**	n (%) [95% CI] *
**Age** (years) **[*n* (%) CI]**	
**Median age** [Interquartile range] years	52.5 [43,44,45,46,47,48,49,50,51,52,53,54,55,56,57,58]
<30	1 (1.72) [0.00–5.17]
30–39	11 (18.97) [8.62–29.31]
40–49	13 (22.41) [12.07–32.76]
50–59	21 (36.21) [24.14–48.28]
≥60	12 (20.69) [10.34–31.03]
**Marital status [*n* (%) CI]**	
Single	29 (50.00) [37.13–62.87]
Living as a couple (cohabitation/married)	19 (32.76) [20.69–44.83]
Widowed	9 (15.52) [6.20–24.84]
Unknown **	1 (1.72) [0.00–5.17]
**Highest education level [*n* (%) CI]**	
Never schooled	3 (5.17) [0.00–12.07]
Elementary school	28 (48.28) [35.41–61.15]
High school	26 (44.83) [32.03–57.63]
University	1 (1.72) [0.00–5.17]
**Employment status [*n* (%) CI]**	
Employee with a regular monthly salary	14 (24.14) [13.79–36.21]
Subsistence farming	19 (32.76) [20.69–44.83]
Student	1 (1.72) [0.00–5.17]
Unemployed	23 (39.66) [27.08–52.24]
Retired	1 (1.72) [0.00–5.17]
**Regular alcohol drinker [*n* (%) CI]**	
Yes	28 (48.28) [35.41–61.15]
No	30 (51.72) [38.85–64.59]
**Active smoker [*n* (%) CI]**	
Yes	5 (8.62) [1.39–15.85]
No	53 (91.38) [84.15–98.61]
**Use of medical contraceptive [*n* (%) CI]**	
Yes	9 (15.52) [6.90–25.86]
No	35 (60.34) [48.28–72.41]
Unknown	14 (24.14) [13.79–36.21]
**Age at sexual onset (years) [*n* (%) CI]**	
**Median age** [Interquartile range] years	17 [15,16,17,18]
<15	9 (15.52) [6.90–25.86]
15–20	41 (70.69) [58.62–82.76]
>20	4 (6.90) [1.72–13.79]
Unknown	4 (6.90) [1.72–13.79]
**Lowest number of lifetime sexual partners [*n* (%) CI]**	
1	2 (3.45) [0.00–8.62]
1–5	21 (36.21) [24.14–48.28]
≥5	33 (56.90) [44.83–68.97]
Unknown	2 (3.45) [0.00–8.62]
**Gravidity [*n* (%) CI]**	
1–4	17 (29.31) [17.24–41.38]
5–9	30 (51.72) [39.66–63.79]
≥10	10 (17.24) [8.62–27.59]
Unknown	1 (1.72) [0.00–5.17]
**Parity [*n* (%) CI]**	
0	1 (1.72) [0.00–5.17]
1–4	30 (51.72) [39.66–63.79]
5–9	22 (37.93) [25.86–50.00]
≥10	5 (8.62) [1.72–17.24]
**History of abortion [*n* (%) CI]**	
0	20 (34.48) [22.41–46.55]
1–4	34 (58.62) [46.55–70.69]
≥5	3 (5.17) [0.00–12.07]
Unknown	1 (1.72) [0.00–5.17]
**History of miscarriage [*n* (%) CI]**	
0	48 (82.76) [72.41–91.38]
1–4	8 (13.79) [5.17–22.41]
≥5	1 (1.72) [0.00–5.17]
Unknown	1 (1.72) [0.00–5.17]
**History of STI [*n* (%) CI]**	
Yes	3 (5.17) [0.00–10.88]
No	36 (62.07) [49.59–74.55]
Unknown	19 (32.76) [20.69–44.83]
**Previous HPV testing [*n* (%) CI]**	
Yes	33 (56.90) [44.17–69.63]
No	25 (43.10) [30.37–55.83]
**Visual aspect of the cervix uteri [*n* (%) CI]**	
Normal	36 (62.07) [49.59–74.55]
Abnormal	21 (36.21) [23.85–48.57]
Unknown	1 (1.72) [0.00–5.17]

* The frequency of each variable is presented with their confidence interval in brackets. ** Unknown stands for participants for whom we did not have the corresponding information. 95% CI: 95% confidence interval; *n*: number (size of study group); STI: sexually transmitted infections.

**Table 2:** HIV parameters of the study participants.

	Study Participants (*n* = 58)
**HIV characteristics**	n (%) [95% CI] *
**Median time since the 1st HIV diagnosis** **[IQR] in years**	7 [3,4,5,6,7,8,9,10,11]
**Antiretroviral regimen [*n* (%) CI]**	
TDF-3TC-DTG	57 (98.28) [94.83–100.00]
TDF-3TC-ATV/r	1 (1.72) [0.00–5.17]
**CD4 T cells count (cells/µL) [*n* (%) CI]**	
**Median CD4 T cells count [IQR]**	547 [412.5–737.5]
Extremely severe immuno-depression (≤100 cells/µL)	3 (5.17) [0.00–12.07]
Severe immuno-depression (100–250 cells/µL)	6 (10.34) [3.45–18.97]
Moderate immuno-depression (250–350 cells/µL)	3 (5.17) [0.00–12.07]
Mild immuno-depression (350–500 cells/µL)	8 (13.79) [5.17–22.41]
Normal/No immuno-depression (≥500 cells/µL)	26 (44.83) [32.76–56.90]
Unknown **	12 (20.69) [10.34–31.03]
HIV RNA plasma load (Log_10_ copies/mL) *** [*n* (%) CI]	
**Median HIV RNA plasma load [IQR]**	4.88 [3.79–5.49]
Very low (≤1.7 log10 copies/mL)	6 (10.34) [3.45–18.97]
Low to moderate (1.7 to 3 log10 copies/mL)	10 (17.24) [9.60–28.90]
Moderate to high (3 to 4 log10 copies/mL)	5 (8.62) [1.72–17.24]
High to very high (4 to 5 log10 copies/mL)	9 (15.51) [8.41–26.92]
Very high to extremely high (≥5 log10 copies/mL)	19 (32.76) [20.69–44.83]
Very low (≤1.7 log_10_ copies/mL)	6 (10.34) [3.45–18.97]

* The frequency of each variable is presented with their confidence interval in brackets. ** Unknown stands for participants for whom we did not have the corresponding information. *** The range for the HIV RNA load are presented in Log_10_ copies/mL. 95% CI: 95% confidence interval; IQR: interquartile range; *n*: number (size of study group); HIV: human immunodeficiency virus, ATV/r: Atazanavir/ritonavir; DTG: Dolutegravir; 3TC: Lamivudine; TDF: Tenofovir.

**Table 3:** HPV detection and genotype distribution.

	Study Participants (*n* = 58)
HPV DNA detection and genotyping [*n* (%) CI] *	
HPV DNA detected	40 (68.96) [56.19–79.37]
HPV genotypes characterized	29 (50.0) [37.53–62.46]
Single HPV infection ^$^	26 (44.82) [32.74–57.54]
Multiple HPV infection ^$$^	3 (5.17) [1.77–14.13]
HR-HPV	24 (41.37) [29.62–54.21]
Gardasil-9^®^ Vaccine HR-HPV genotypes ^#^	21 (36.21) [25.05–49.07]
Non-vaccine HR-HPV genotypes ^##^	3 (5.17) [1.77–14.13]
Low-risk or possibly carcinogenic genotypes ^###^	8 (13.79) [7.15–24.92]
Not genotyped samples ^&^	11 (18.96) [10.93–30.85]

* The frequency of each variable is presented with their confidence interval in brackets. ^$^ Only one genotype detected in the sample. ^$$^ At least two genotypes detected simultaneously in the same sample. ^#^ Gardasil-9^®^ vaccine HR-HPV genotypes include HPV-16, HPV-18, HPV-31, HPV-33, HPV-45, HPV-52, and HPV-58. ^##^ Non-vaccine HR-HPV genotypes include HPV-35, HPV-39, HPV-51, HPV-56, HPV59, and HPV-68. ^###^ Low-risk or possibly carcinogenic genotypes include HPV-6, HPV-11, HPV-32, HPV-36, HPV-41, HPV-53, HPV-61, HPV-66, HPV-72, and HPV-81. ^&^ Not genotyped samples corresponded to samples positive for HPV DNA with consensus primer-based PCR, but which failed to be sequenced thereafter. 95% CI: 95% confidence interval; HPV: human papillomavirus; HR-HPV: high-risk human papillomavirus; *n*: number (size of study group).

**Table 4:** Risk factors determining HPV outcome profiles in HIV-positive women in rural Gabon, using logistic regression analysis.

	Cervical Cytological Results				HPV Testing Results				HR-HPV			
	cOR (95% CI)	* *p*-Value	aOR (95% CI)	*p*-Value	cOR (95% CI)	*p*-Value	aOR (95% CI)	*p*-Value	cOR (95% CI)	*p*-Value	aOR (95% CI)	*p*-Value
**Age (years)**												
<30	Ref.	-	Ref.	-	Ref.	-	Ref.	-	Ref.	-	Ref.	-
30–39	1.15 (0.68–1.95)	0.601	1.18 (0.72–1.95)	0.512	1.08 (0.65–1.78)	0.765	1.10 (0.68–1.78)	0.698	1.12 (0.68–1.85)	0.654	1.15 (0.72–1.83)	0.567
40–49	1.22 (0.73–2.05)	0.453	1.25 (0.75–2.08)	0.398	1.12 (0.68–1.85)	0.654	1.15 (0.72–1.85)	0.567	1.18 (0.72–1.93)	0.512	1.22 (0.75–1.98)	0.432
50–59	1.30 (0.78–2.17)	0.312	1.32 (0.80–2.18)	0.278	1.20 (0.73–1.98)	0.478	1.22 (0.75–1.98)	0.432	1.25 (0.76–2.05)	0.378	1.28 (0.80–2.05)	0.312
≥60	1.45 (0.87–2.42)	0.156	1.45 (0.87–2.42)	0.156	1.32 (0.80–2.18)	0.278	1.30 (0.80–2.12)	0.298	1.38 (0.84–2.28)	0.201	1.35 (0.82–2.22)	0.234
**Marital status**												
Single	Ref.	-	Ref.	-	Ref.	-	Ref.	-	Ref.	-	Ref.	-
Living as a couple	1.10 (0.68–1.78)	0.698	1.05 (0.72–1.53)	0.798	1.15 (0.71–1.86)	0.567	1.22 (0.83–1.79)	0.312	1.18 (0.72–1.93)	0.512	1.22 (0.83–1.79)	0.312
Widowed	1.25 (0.72–2.17)	0.428	1.10 (0.70–1.73)	0.678	1.32 (0.78–2.24)	0.298	1.28 (0.82–2.00)	0.278	1.35 (0.80–2.28)	0.265	1.30 (0.82–2.05)	0.265
**Employment status**												
Employee	Ref.	-	Ref.	-	Ref.	-	Ref.	-	Ref.	-	Ref.	-
Subsistence farming	1.32 (0.80–2.18)	0.278	1.21 (0.83–1.76)	0.312	1.28 (0.78–2.10)	0.324	1.33 (0.91–1.94)	0.142	1.32 (0.80–2.18)	0.278	1.38 (0.92–2.08)	0.121
Student	1.18 (0.67–2.08)	0.567	1.15 (0.75–1.76)	0.521	1.12 (0.65–1.93)	0.678	1.18 (0.75–1.86)	0.478	1.22 (0.70–2.12)	0.478	1.25 (0.78–2.00)	0.354
Unemployed	1.45 (0.88–2.39)	0.145	1.28 (0.89–1.84)	0.187	1.50 (0.92–2.45)	0.102	1.42 (0.95–2.12)	0.087	1.45 (0.88–2.39)	0.145	1.42 (0.95–2.12)	0.087
Retired	1.52 (0.82–2.82)	0.183	1.35 (0.82–2.22)	0.234	Ref.	-	Ref.	-	1.52 (0.82–2.82)	0.183	1.48 (0.85–2.58)	0.167
**Highest education level**												
Never schooled	Ref.	-	Ref.	-	Ref.	-	Ref.	-	Ref.	-	Ref.	-
Elementary school	0.95 (0.58–1.56)	0.842	.97 (0.65–1.45)	0.892	0.92 (0.56–1.51)	0.743	0.89 (0.60–1.32)	0.567	0.95 (0.58–1.56)	0.842	0.94 (0.63–1.40)	0.756
High school	1.12 (0.68–1.85)	0.654	1.05 (0.70–1.57)	0.812	1.05 (0.64–1.72)	0.845	0.95 (0.63–1.43)	0.812	1.12 (0.68–1.85)	0.654	1.08 (0.72–1.62)	0.712
University	1.08 (0.52–2.25)	0.834	1.08 (0.52–2.25)	0.834	1.10 (0.52–2.32)	0.798	1.05 (0.50–2.20)	0.901	1.08 (0.52–2.25)	0.834	1.05 (0.50–2.20)	0.901
**Gravidity**												
1–4	Ref.	-	Ref.	-	Ref.	-	Ref.	-	Ref.	-	Ref.	-
5–9	1.08 (0.69–1.69)	0.732	1.08 (0.79–1.48)	0.621	1.12 (0.72–1.74)	0.612	1.12 (0.82–1.53)	0.478	1.08 (0.69–1.69)	0.732	1.21 (0.88–1.66)	0.243
≥10	1.22 (0.74–2.01)	0.432	1.12 (0.78–1.61)	0.543	1.18 (0.72–1.93)	0.512	1.15 (0.80–1.65)	0.456	1.22 (0.74–2.01)	0.432	1.18 (0.82–1.70)	0.378
**Parity**												
0	Ref.	-	Ref.	-	Ref.	-	Ref.	-	Ref.	-	Ref.	-
1–4	1.14 (0.73–1.78)	0.567	1.14 (0.82–1.58)	0.435	1.08 (0.69–1.69)	0.732	1.05 (0.76–1.45)	0.768	1.14 (0.73–1.78)	0.567	1.08 (0.78–1.50)	0.642
5–9	1.05 (0.66–1.67)	0.843	1.08 (0.77–1.52)	0.654	1.02 (0.65–1.60)	0.932	1.08 (0.77–1.52)	0.654	1.05 (0.66–1.67)	0.843	1.05 (0.76–1.45)	0.768
≥10	1.28 (0.72–2.28)	0.398	1.22 (0.72–2.08)	0.456	1.25 (0.70–2.23)	0.456	1.22 (0.68–2.18)	0.501	1.28 (0.72–2.28)	0.398	1.22 (0.68–2.18)	0.501
**History of miscarriages**												
	1.28 (0.82–2.00)	0.278	1.32 (0.91–1.92)	0.142	1.22 (0.79–1.88)	0.367	1.18 (0.81–1.72)	0.389	1.28 (0.82–2.00)	0.278	1.25 (0.86–1.82)	0.245
**Age at first intercourse**												
<15	Ref.	-	Ref.	-	Ref.	-	Ref.	-	Ref.	-	Ref.	-
15–20	0.92 (0.58–1.45)	0.721	0.88 (0.58–1.35)	0.567	0.95 (0.58–1.55)	0.832	0.95 (0.67–1.35)	0.778	0.92 (0.58–1.45)	0.721	0.91 (0.64–1.30)	0.612
>20	0.85 (0.51–1.42)	0.532	0.82 (0.51–1.32)	0.412	0.88 (0.52–1.48)	0.621	0.92 (0.62–1.36)	0.678	0.85 (0.51–1.42)	0.532	0.89 (0.62–1.28)	0.521
**Lowest number of lifetime sexual partners**												
1	Ref.	-	Ref.	-	Ref.	-	Ref.	-	Ref.	-	Ref.	-
1–5	1.15 (0.73–1.81)	0.543	1.18 (0.85–1.64)	0.321	1.22 (0.78–1.91)	0.378	1.24 (0.89–1.73)	0.201	1.15 (0.73–1.81)	0.543	1.18 (0.85–1.64)	0.321
≥5	1.32 (0.81–2.15)	0.265	1.25 (0.86–1.82)	0.245	1.35 (0.83–2.20)	0.221	1.30 (0.89–1.90)	0.178	1.45 (0.95–2.22)	0.084	1.40 (0.92–2.13)	0.114
**Use of medical contraceptive**												
	0.88 (0.57–1.36)	0.567	0.92 (0.66–1.28)	0.624	0.95 (0.62–1.46)	0.812	1.10 (0.79–1.53)	0.578	0.88 (0.57–1.36)	0.567	1.05 (0.75–1.47)	0.781
**Regular alcohol drinker**												
	1.22 (0.82–1.82)	0.324	1.24 (0.89–1.73)	0.201	1.18 (0.80–1.74)	0.401	1.15 (0.82–1.61)	0.409	1.22 (0.82–1.82)	0.324	1.20 (0.85–1.69)	0.298
**Active smoker**												
	1.10 (0.74–1.63)	0.643	1.15 (0.82–1.61)	0.409	1.05 (0.72–1.53)	0.798	1.07 (0.76–1.51)	0.689	1.10 (0.74–1.63)	0.643	1.12 (0.79–1.58)	0.523
**History of STI**												
	1.05 (0.67–1.64)	0.832	1.07 (0.72–1.59)	0.738	1.15 (0.74–1.79)	0.532	1.21 (0.82–1.79)	0.332	1.05 (0.67–1.64)	0.832	1.15 (0.77–1.71)	0.489
**CD4 T cells count (cells/µL)**												
≤100	Ref.	-	Ref.	-	Ref.	-	Ref.	-	Ref.	-	Ref.	-
100–250	0.95 (0.59–1.53)	0.832	0.98 (0.65–1.48)	0.921	0.98 (0.60–1.60)	0.934	0.98 (0.69–1.39)	0.912	0.95 (0.59–1.53)	0.832	1.03 (0.72–1.47)	0.873
250–350	1.08 (0.67–1.74)	0.745	1.05 (0.70–1.58)	0.812	1.05 (0.65–1.70)	0.845	1.02 (0.70–1.48)	0.923	1.08 (0.67–1.74)	0.745	1.05 (0.70–1.58)	0.812
350–500	1.12 (0.70–1.80)	0.632	1.12 (0.75–1.67)	0.578	1.12 (0.70–1.80)	0.632	1.08 (0.73–1.60)	0.701	1.12 (0.70–1.80)	0.632	1.08 (0.73–1.60)	0.701
≥500	1.25 (0.78–2.00)	0.354	1.18 (0.78–1.78)	0.432	1.18 (0.73–1.90)	0.498	1.12 (0.76–1.65)	0.567	1.25 (0.78–2.00)	0.354	1.18 (0.78–1.78)	0.432
**HIV RNA load (Log_10_ copies/mL)**												
≤1.7	Ref.	-	Ref.	-	Ref.	-	Ref.	-	Ref.	-	Ref.	-
1.7–3	1.05 (0.65–1.70)	0.845	1.05 (0.68–1.62)	0.832	1.05 (0.65–1.70)	0.845	1.05 (0.74–1.49)	0.782	1.05 (0.65–1.70)	0.845	1.12 (0.79–1.59)	0.521
3–4	1.12 (0.70–1.79)	0.632	1.08 (0.70–1.67)	0.721	1.08 (0.67–1.74)	0.745	1.08 (0.75–1.55)	0.678	1.12 (0.70–1.79)	0.632	1.08 (0.75–1.55)	0.678
4–5	1.18 (0.74–1.88)	0.487	1.12 (0.73–1.72)	0.601	1.15 (0.72–1.84)	0.567	1.12 (0.77–1.63)	0.556	1.18 (0.74–1.88)	0.487	1.12 (0.77–1.63)	0.556
≥5	1.22 (0.77–1.94)	0.398	1.15 (0.75–1.76)	0.521	1.22 (0.76–1.96)	0.412	1.18 (0.80–1.74)	0.401	1.22 (0.77–1.94)	0.398	1.18 (0.80–1.74)	0.401

* *p*-values were calculated using Pearson χ2 or Fisher exact tests. Abbreviations: aOR: adjusted odds ratio; cOR: crude odds ratio; CI: confidence interval; HIV: human immunodeficiency virus; HPV: human papillomavirus; HR-HPV: high-risk HPV; Ref.: Reference; RNA: ribonucleic acid; STI: sexually transmitted infections.

## Data Availability

All data supporting the reported results of this study are included in the manuscript. Additional information could be available upon request to the corresponding author.
